# The golden death bacillus *Chryseobacterium nematophagum* is a novel matrix digesting pathogen of nematodes

**DOI:** 10.1186/s12915-019-0632-x

**Published:** 2019-02-28

**Authors:** Antony P. Page, Mark Roberts, Marie-Anne Félix, Derek Pickard, Andrew Page, William Weir

**Affiliations:** 1 Institute of Biodiversity, Animal Health and Comparative Medicine, University of Glasgow, Scotland, UK; 2 School of Veterinary Medicine, University of Glasgow, Scotland, UK; 3 Institute of Biology of the Ecole Normale Supérieure (IBENS), Paris, France; 4 Wellcome Trust Sanger Institute, Hinxton, Cambridge, UK

**Keywords:** Nematode parasites, *Chryseobacterium*, Matrix digesting, Collagenase, Chitinase, Biological control agent

## Abstract

**Background:**

Nematodes represent important pathogens of humans and farmed animals and cause significant health and economic impacts. The control of nematodes is primarily carried out by applying a limited number of anthelmintic compounds, for which there is now widespread resistance being reported. There is a current unmet need to develop novel control measures including the identification and characterisation of natural pathogens of nematodes.

**Results:**

Nematode killing bacilli were isolated from a rotten fruit in association with wild free-living nematodes. These bacteria belong to the *Chryseobacterium* genus (golden bacteria) and represent a new species named *Chryseobacterium nematophagum*. These bacilli are oxidase-positive, flexirubin-pigmented, gram-negative rods that exhibit gelatinase activity. *Caenorhabditis elegans* are attracted to and eat these bacteria. Within 3 h of ingestion, however, the bacilli have degraded the anterior pharyngeal chitinous lining and entered the body cavity, ultimately killing the host. Within 24 h, the internal contents of the worms are digested followed by the final digestion of the remaining cuticle over a 2–3-day period. These bacteria will also infect and kill bacterivorous free-living (L1-L3) stages of all tested parasitic nematodes including the important veterinary Trichostrongylids such as *Haemonchus contortus* and *Ostertagia ostertagi*. The bacteria exhibit potent collagen-digesting properties, and genome sequencing has identified novel metalloprotease, collagenase and chitinase enzymes representing potential virulence factors.

**Conclusions:**

*Chryseobacterium nematophagum* is a newly discovered pathogen of nematodes that rapidly kills environmental stages of a wide range of key nematode parasites. These bacilli exhibit a unique invasion process, entering the body via the anterior pharynx through the specific degradation of extracellular matrices. This bacterial pathogen represents a prospective biological control agent for important nematode parasites.

**Electronic supplementary material:**

The online version of this article (10.1186/s12915-019-0632-x) contains supplementary material, which is available to authorized users.

## Background

Parasitic nematodes inflict a major burden on public health and on the farming industry worldwide. It is estimated that more than one billion people are suffering from soil-transmitted nematode infestations, such as hookworm infection, Ascariasis and Trichuriasis. These parasites cause significant lifelong morbidity [1]. The veterinary impact of disease caused by nematodes is enormous, with an estimated annual economic loss of $2 billion to livestock production in North America alone [2]. Moreover, the losses in crop yields caused by plant-parasitic nematodes are estimated to be as much as $125 billion per year [3].

Several classes of highly effective anthelmintic molecules were introduced, first for veterinary purposes, such as the benzimidazoles (e.g. albendazole) and the imidazothiazoles (e.g. levamisole) in the 1960s, the macrocyclic lactones (e.g. ivermectin and moxidectin) in the 1980s and the novel amino-acetonitrile derivative monepantel in 2009 [4]. However, drug resistance has arisen unexpectedly rapidly, mainly in South Africa, USA and Australia with for example, the first cases of resistance to benzimidazoles reported within 5 years of drug release [5]. Resistance to ivermectin and other macrocyclic lactones has now also been reported in numerous countries with intensive farming activities. The problem of drug resistance strikes the global sheep industry particularly hard, with resistance prevalence often exceeding 50% of all infections [6]. Moreover, anthelmintic resistance is irreversible [7]. It is predicted that the high levels of resistance currently observed in veterinary parasites will ultimately develop in the soil-transmitted nematodes of humans [6]. Despite decades of research, only two species-specific vaccines with limited application are currently available [8]. With the above control limitations, there is a pressing need to develop new methods of nematode control, particularly for the ubiquitous Trichostrongylid parasites of livestock.

The Trichostrongyles have obligate environmental developmental stages (egg-L3), and while grazing management methods can to some extent limit parasite exposure, an important unexploited means of control is the application of natural predators of these free-living stages. One such method is the nematode-trapping fungus *Duddington flagrans* that has been shown to reduce the pasture levels of infective larvae [9]. In the case of plant parasitic nematodes, biocontrol through the application of species-specific bacterial pathogens, such as *Pasteuria penetrans*, is well established [10] and is now commercially available. A current unmet need is to discover and develop new biocontrol measures that will reduce the larval infection of pasture with Trichostrongyles to a level that avoids both clinical and sub-clinical disease in grazing livestock. Such control measures will help curtail resistance, thereby preserving the available anthelmintics for the treatment of diseased animals.

The free-living nematode *Caenorhabditis elegans* is an excellent genetically tractable model that has been used extensively to study nematode pathogens, the majority of which are, however, only effective against *Caenorhabditis* species and not against parasitic species [11]. In this study, we describe the isolation and characterisation of a novel *Chryseobacterium* pathogen with great potential for controlling key nematode infections of veterinary importance. *Chryseobacterium* spp. are gram-negative rods found ubiquitously in the environment with certain species being reported as having unusual matrix digesting properties [12].

## Results

In this study, we searched the environment for natural nematode pathogenic bacteria in association with wild *Caenorhabditis* nematodes. The bacterial strain JUb129 was isolated from the free-living bacterivorous nematode *Caenorhabditis briggsae* from a rotten apple in Paris, France (NCBI BioSample SAMN09925763) [13]. JUb129 was also found to display unusual pathogenic activity against *C. elegans* [14]. The JUb275 bacteria were subsequently isolated (December 2016) from *Caenorhabditis briggsae* found on a rotten fig in Bangalore, India (NCBI BioSample SAMN09925764) [15]. Both these species were found to be highly pathogenic to *C. briggsae*. Additional nematode-associated *Chryseobacterium* and related *Flavobacterium* species were obtained from further environmental samples together with a plant root, amphibian and a chicken-associated *Chryseobacterium* species. All isolates were tested for nematode killing properties against *C. elegans*. Of all the isolates tested, only JUb129 and JUb275 were found to kill *C. elegans* (Table 1).

**Table 1:** Source of *Chryseobacterium* and ability to kill *C. elegans*

Strain ID	Strain name	Source	Nematode killing
JUb129	*Chryseobacterium nematophagum*	Orsay France, rotten apple	Yes
JUb275	*Chryseobacterium nematophagum*	Bangalore, India, rotten fig	Yes
JUb270	*Chryseobacterium shigense*	Paris, France	No
JUb232	*Chryseobacterium indoltheticum*	Plurien, France, plums	No
JUb171	*Flavobacterium banpakuense*	Orsay, France, apple	No
JUb166	*Flavobacterium banpakuense*	Orsay, France, apple	No
JUb044	*Chryseobacterium* sp.	Santeuil, France, compost	No
JUb043	*Flavobacterium* sp.	Santeuil, France, apple	No
JUb022	*Flavobacterium* sp.	Paris, France, flower stem	No
JUb007	*Chryseobacterium* sp.	Le Perreux, France, compost	No
JUb001	*Flavobacterium* sp.	Le Perreux, France, compost	No
100 T	*Chryseobacterium gallinarium*	Saxony, Germany, chicken	No
C26T	*Chryseobacterium contaminans*	Alabama USA, rhizosphere soil	No
–	*Chryseobacterium indologenes*	Glasgow, UK, toad	No

### Microbiology

Our bacteriological characterisation indicated that JUb129 and JUb275 belong to the *Chryseobacterium* genus. In common with other *Chryseobacterium*, both strains are catalase and oxidase-positive, aerobic gram-negative rods that grow on solid media to produce golden, mucoid colonies that have a pungent odour. The golden colour was shown to be due to the production of a flexirubin-type pigment (Additional file 1). Both JUb129 and JUb275 were found to grow optimally at 30 °C (neither grows at 37 °C) on agar plus 5% sheep blood, or tryptone soy agar plus 5% sheep blood, and also grew well but less optimally on LB agar. In liquid media, growth was more efficient in SOB media than LB media. In API 20E and API 20NE strips, the strains gave positive results for gelatin, esculin and *N*-acetylglucosamine (Additional files 1 and 2). The API 20E test result gave the closest identification to *Chryseobacterium indologenes* (86.3%).

### Phylogenetic analysis

**Fig. 1:**
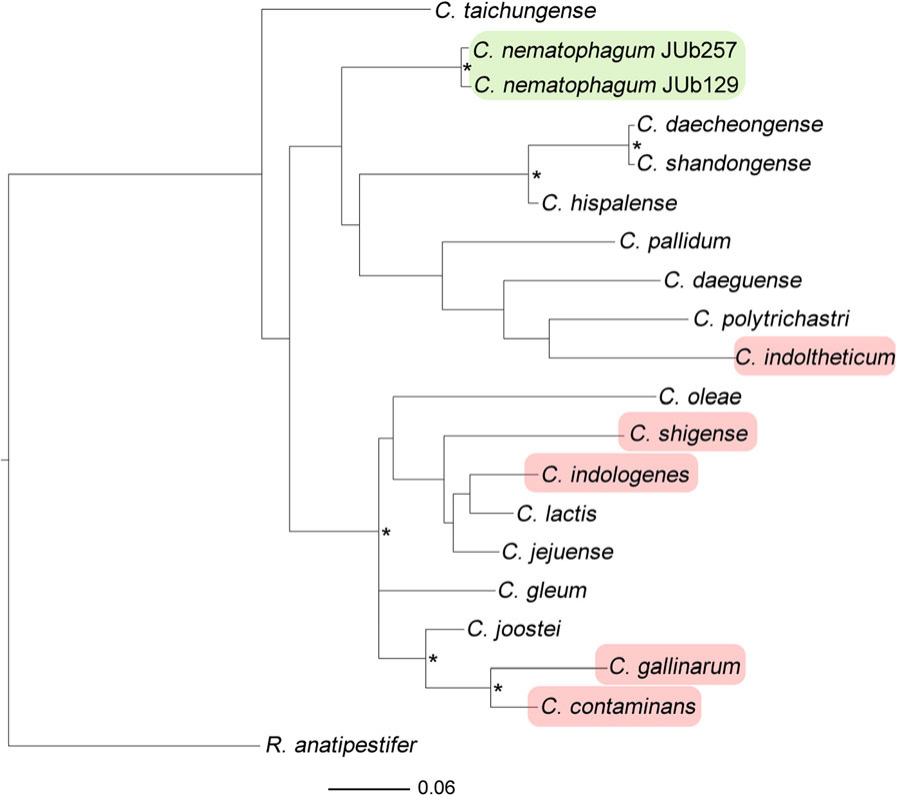
Phylogenetic analysis of *C. nematophagum* isolates. A maximum likelihood tree was constructed using the 16S rRNA gene sequences of a number of *Chryseobacterium* species, including *C. nematophagum* (JUb129 and JUb275). The tree was rooted using a *Reimerella anatipestifer* 16S sequence. Bacterial species with the nematode-killing phenotype are shown in green, while those demonstrated not to have the phenotype are shown in red. Nodes with > 80% bootstrap support are indicated with an asterisk

Confirmation of genus designation was obtained following whole-genome sequencing of the 4.5 Mb genomes of both the JUb129 and JUb275 isolates. The loci encoding the 16S SSU rRNA genes were identified and the sequences used to construct a phylogenetic tree (Fig. 1). The genomes of both JUb129 and JUb275 encode multiple copies of the 16S rRNA gene. JUb275 contained six identical copies of the gene while a single-nucleotide polymorphism was present in one of the six copies in JUb129. The JUb129 consensus sequence differed from the JUb275 sequence by only three nucleotides, revealing that the isolates are very similar but distinct from one other. The tree was rooted using the 16S sequence of a member of a different genus within the family Flavobacteriaceae, *Reimerella anatipestifer*. The JUb129 and JUb275 sequences were found to fall within the *Chryseobacterium* clade, indicating these isolates represent a novel species belonging to the *Chryseobacterium* genus, closely related to other environmental bacteria such as *C. pallidum* [16], *C. indoltheticum* [17] and *C. hispalense*. Consequently, we have named this new species *Chryseobacterium nematophagum* (from Greek crysos meaning golden and phago meaning devour; the golden nematode-devouring bacterium).

### Infection and killing of *Caenorhabditis elegans*

**Fig. 2:**
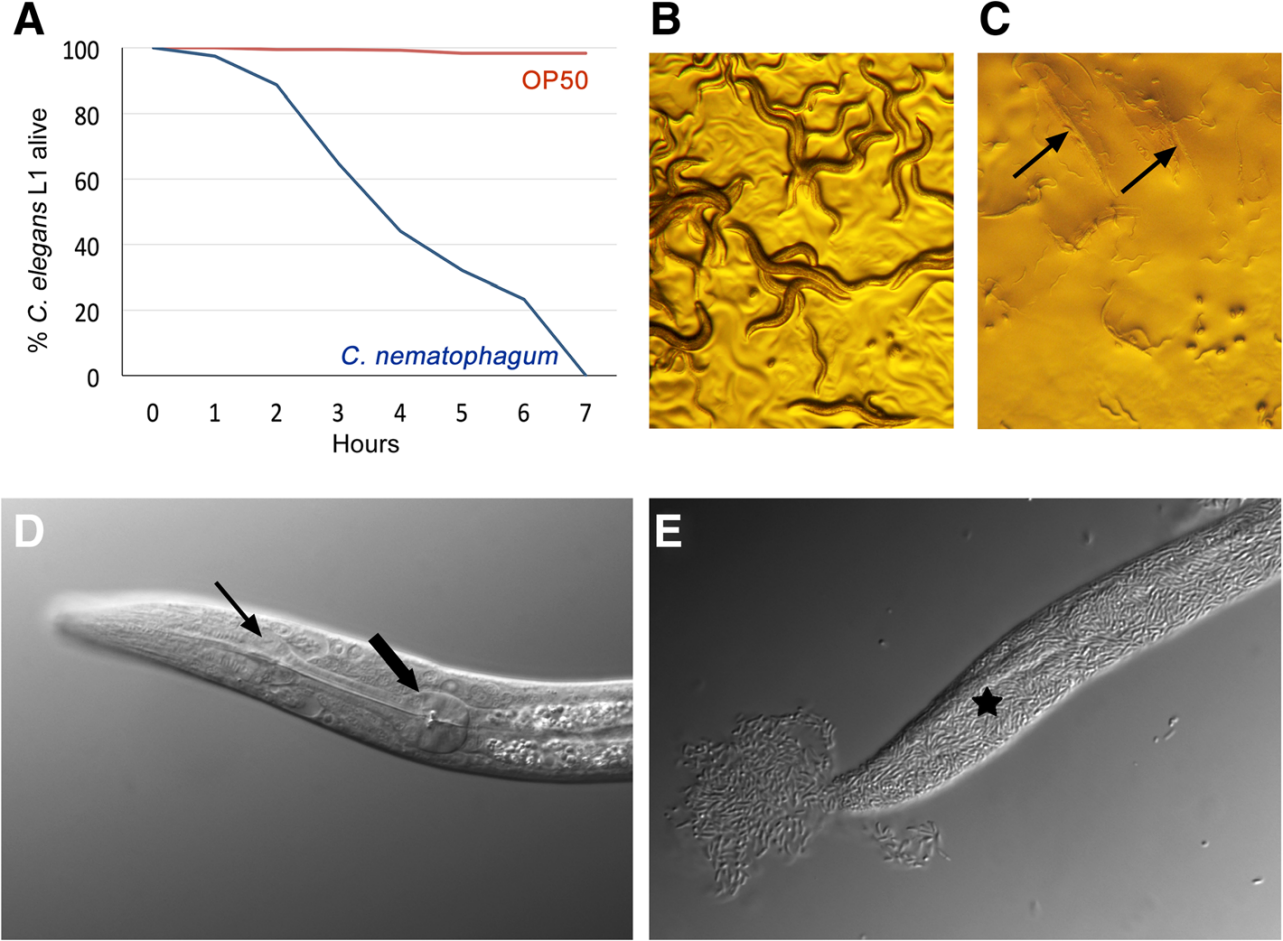
Time-course of *C. elegans* killing by *C. nematophagum*. **a** Timecourse of 241 L1 *C. elegans* survival (% alive) in the presence of OP50 (red), compared to 193 L1 *C. elegans* in the presence of *C. nematophagum* (blue). **b** *C. elegans* mixed population exposed to *Chryseobacterium gallinarum* for 48 h (× 40). **c** *C. elegans* mixed population exposed to *C. nematophagum* for 48 h, dead bacteria filled carcasses arrowed (× 40) **d** *C. elegans* L1 cultured in OP50 for 48 h (× 640); small arrow denotes anterior pharyngeal bulb and large arrow the posterior pharyngeal bulb. **e** *C. elegans* L1 cultured for 48 h with *C. nematophagum*, only structureless bacterial-filled carcass (star) remains (× 640)

Infection experiments were carried out on staged populations of *C. elegans* wild type strain N2, following bleach treatment to purify and sterilise embryos. Embryos were hatched to L1 overnight in M9 buffer, and synchrony of L1 larvae was initiated by feeding on either *Escherichia coli* OP50-1 or JUb275. Synchronised L2, L3 and L4 s were all collected from OP50-1 fed L1s. More than 50% of the L1 population were killed within 3 to 4 h of contact with complete killing noted by 7 h of exposure (Fig. 2a). All larvae generally became immotile with only slight head movements following 1 h of exposure to these bacilli. Similar death rates were found for L2, L3 and L4 s with greater than 50% killing occurring between 2 and 4 h (Additional file 3). Following exposure to bacteria for 48 h, only outline traces of the larvae, representing the undigested cuticles, were present on plates (Fig. 2c and e) whereas the corresponding OP50-1 (Fig. 2d) or *Chryseobacterium gallinarum* (Fig. 2b) fed nematodes had developed further and were thriving on the bacterial food source. Mixing experiments were setup between the normal *C. elegans* food source, OP50-1, and the pathogen *C. nematophagum* (Additional file 4). A very low infectious dose of *C. nematophagum* (200 cfu) mixed with a dense population of OP50-1 (3.8 × 10^7^ cfu) was sufficient to kill 100% of L1s over a 24-h exposure period (Additional file 4).

A common feature associated with many of the previously described *C. elegans* bacterial pathogens is the fact that the nematodes sense and are repelled from the bacterial lawns, as in the case of *Pseudomonas fluorescens* [18] and *Serratia marcescens* [19]. It is significant to note that *C. elegans* are not repelled by *C. nematophagum*, but are attracted to, and remain on the bacterial lawns where they actively ingest the bacteria (Additional file 5).

A wide range of stock and environmentally derived isolates of *Chryseobacterium* were tested for activity against *C. elegans* (Table 1), all of which lacked the unique *C. elegans* killing properties of JUb129 and JUb275. An example of this is *C. gallinarum* (Fig. 2b), isolated from a chicken, which instead provides a nutritional food source that allows full development of *C. elegans*.

### Pharyngeal invasion by *C. nematophagum*

**Fig. 3:**
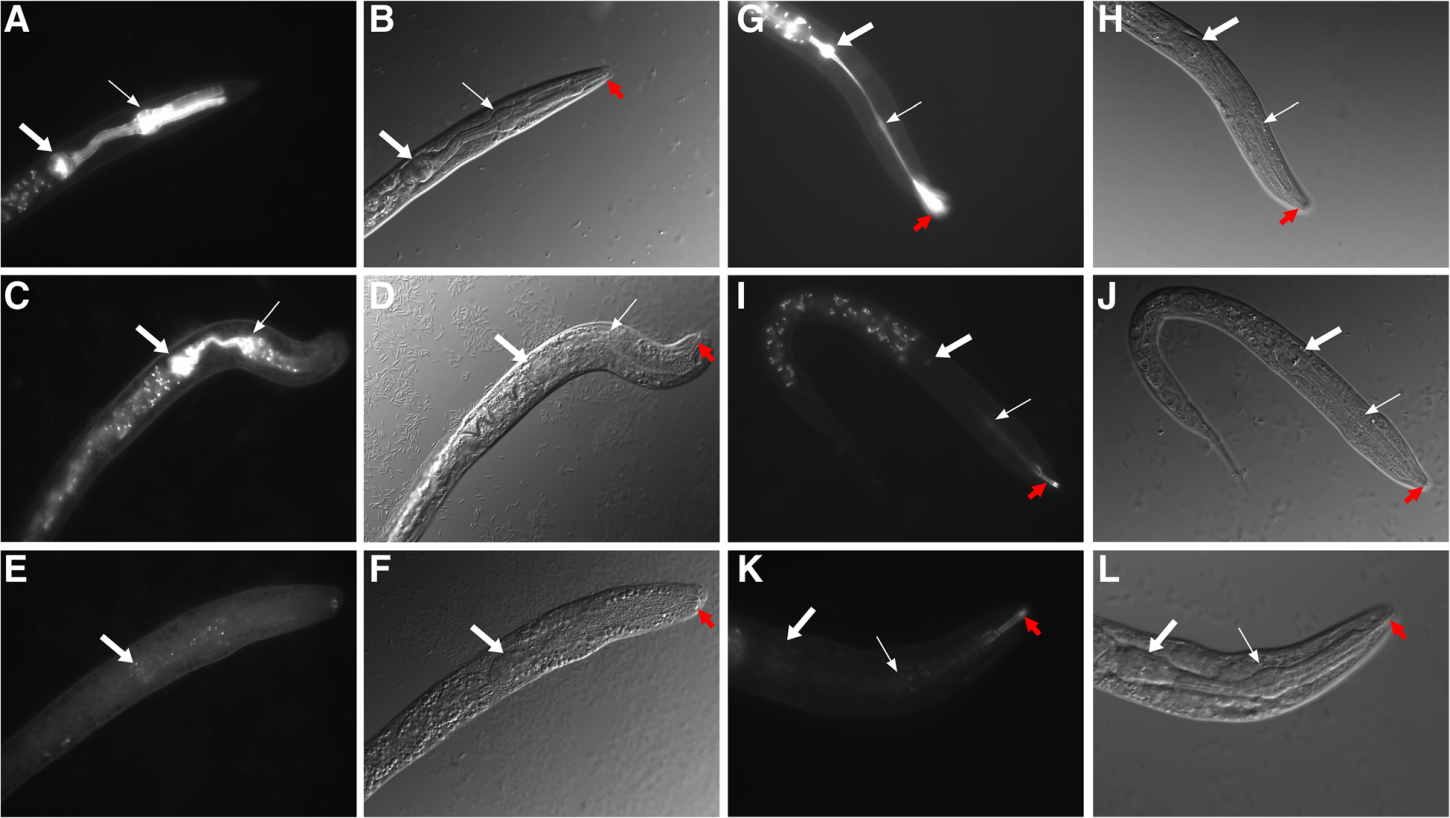
Progressive destruction of the *C. elegans* pharynx by *C. nematophagum.* L1 stage *C. elegans myo2::rfp* (pharyngeal muscle marker) expressing strain VS21 were exposed to *C. nematophagum* for 1 h (**a** and **b**), 3 h (**c** and **d**) and 6 h (**e** and **f**). L1 stage *C. elegans* were stained with eosin Y to highlight chitosan in the pharynx prior to exposure to *C. nematophagum* for 1 h (**g** and **h**), 3 h (**i** and **j**) and 6 h (**k** and **l**). Panels **b**, **d** and **f** Depict DIC images, and **a**, **c** and **e** are the corresponding fluorescent images revealing the progressive breakdown of *myo-2* labelled pharynx. Panels **h**, **j** and **l** depict DIC images, and **g**, **i** and **k** are the corresponding fluorescent images revealing the progressive breakdown of eosin Y-labelled chitosan of the pharynx. Large white arrow, posterior pharyngeal bulb and small white arrow anterior pharyngeal bulb, red arrow highlights the buccal cavity. All images at × 630

Following exposure to the bacterial cultures on plates, *C. elegans* ingests *C. nematophagum*, which in turn multiply in the anterior pharynx and digest the nematode internally, ultimately degrading the external cuticle from the inside (Fig. 2c and e). Using a transgenic marker strain (VS21), encoding *myo-2*::mCherry that highlights the muscular pharynx, there is a progressive destruction of the anterior chitin and collagen-lined pharyngeal procorpus structure, resulting in the breakdown of the anterior pharynx structure and leakage of mCherry into the anterior body cavity (Fig. 3a–f).

To investigate the breakdown of the chitinous lining of the pharynx, the chitosan-specific stain eosin Y [20] was used to highlight this structure in *C. elegans* L1 larvae prior to exposure to *C. nematophagum*. Prior to bacterial exposure and 1 h after exposure, eosin Y delineates the entire pharyngeal and buccal cavity linings (Fig. 3g and h). The pharyngeal staining and hence the chitosan structures are lost following a 3-h exposure to *C. nematophagum*; however, the buccal cavity lining remains intact (Fig. 3i, j, k and l). This ability to breakdown the pharyngeal structure is an unusual attribute, most likely occurring through the action of specific chitinases and collagenase-like metalloproteases.

### Cuticle collagen degradation

**Fig. 4:**
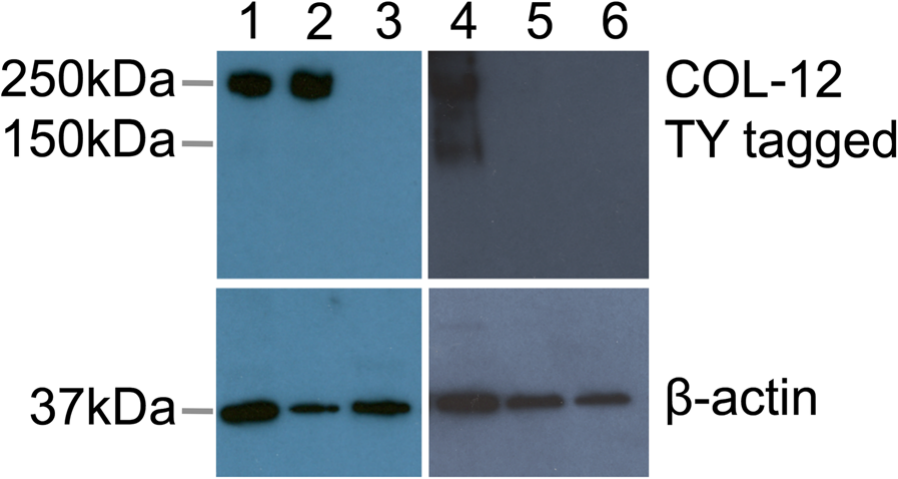
*Chryseobacterium nematophagum* degrade the collagenous matrix of *Caenorhabditis elegans.* COL-12 TY tagged *C. elegans* strains (IA132) were incubated with *C. nematophagum* JUb129 for 24 h (1) 48 h (3) and with OP50 alone control for 48 h (2). IA132 was incubated for 48 h with non-pathogenic *Chryseobacterium indologenes* (4). IA132 adults were pre-cleared of OP50-1 by washing in M9 buffer and culturing on non-seeded plates for 4 h and then incubated for 48 h with *C. nematophagum* JUb129 (5) or JUb275 (6). Twenty adult worms per treatment were extracted and Western blotted and probed with anti-TY tag (upper) and then re-probed anti-β-actin (lower) antibodies

**Fig. 5:**
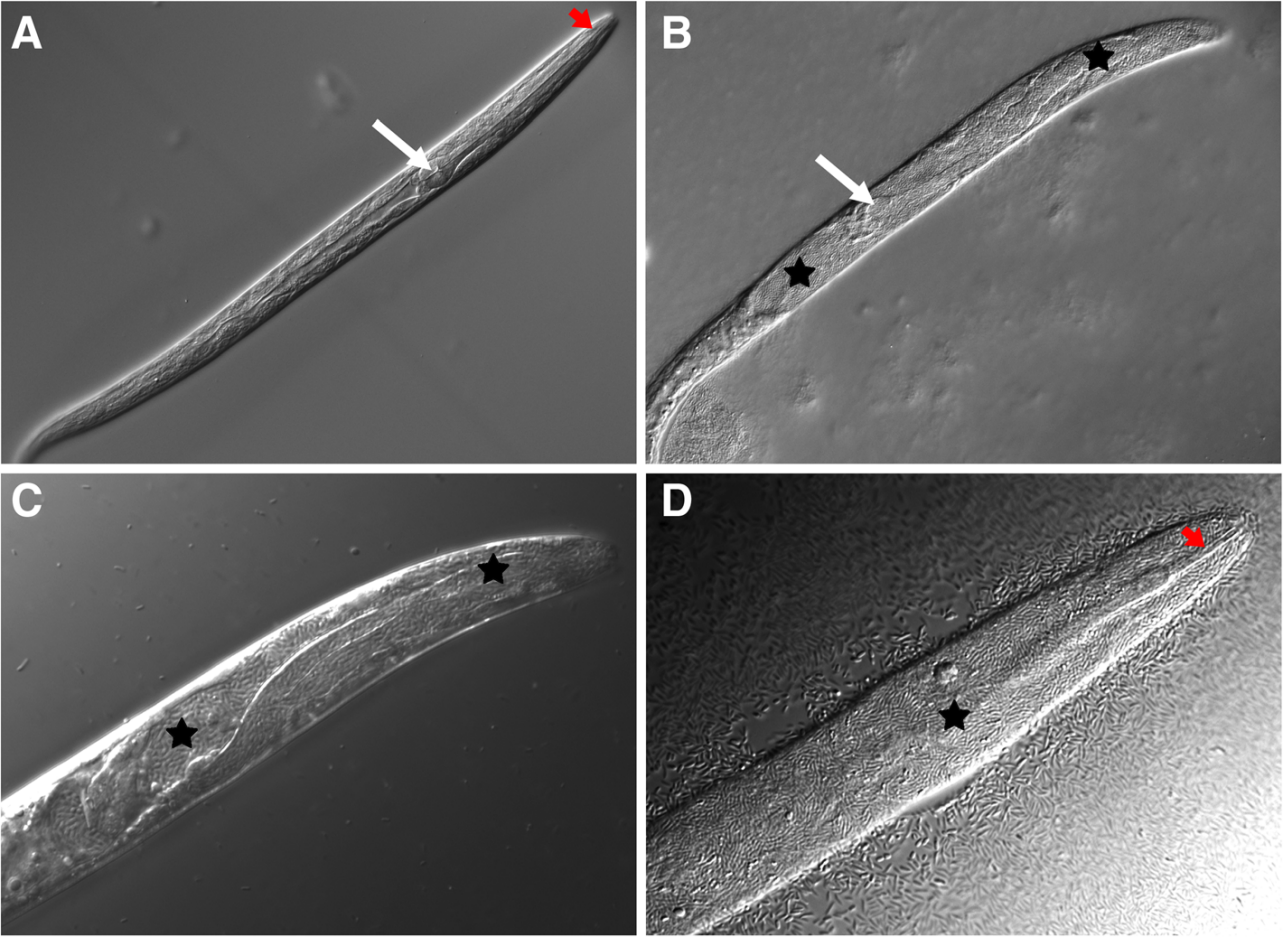
*Haemonchus contortus* (IRE) L2 cultured on OP50 and *C. nematophagum*. **a** L2 cultured on OP50-1 control plates, white arrow pharyngeal bulb and red arrow buccal cavity (× 250). **b** L2 cultured on *C. nematophagum*, anterior body cavity packed with bacilli (black stars) and position of partially digested pharyngeal bulb denoted by white arrow (× 250). **c** L2 cultured on *C. nematophagum*, anterior body cavity packed with bacilli (black stars) (× 630). **d** L2 cultured on *C. nematophagum*, only buccal cavity (red arrow) and cuticle remains and entire body filled with bacilli (black star) (× 63)

The main matrices in nematodes, most notably the cuticle and the pharynx lining, are composed of highly cross-linked collagens [21, 22]. The ability of bacteria to digest these normally insoluble structural components, especially the cuticle, is unusual, and we therefore applied a TY-epitope tagged COL-12 cuticle collagen expressing strain, IA132 [23], to investigate this phenomenon. The adult *C. elegans* transgenic strain IA132 were incubated in the presence of *C. nematophagum* following culture on OP50-1 NGM plates or were cultured on pure OP50-1 plates for 24–48 h prior to preparation of worms for Western blot analysis and probing with anti-TY tag and anti-actin antibodies. The cuticle collagen COL-12 assembles into highly insoluble non-reducible multimeric complexes in excess of 150–250 kDa (Fig. 4, lane 1). These structures are however broken-down following exposure to the *C. nematophagum* for 48 h (Fig. 4, lane 3), and the same samples were subsequently probed with anti β-actin, which revealed this structural protein conversely remained intact (Fig. 4, lane 3), highlighting that this digestion was specific to the COL-12 collagen. To confirm the specificity of this cuticle digestion and to exclude the possibility that OP50-1 was responsible for the cuticle collagen digestion, the following controls were carried out. 1. IA132 were incubated with non-pathogenic *Chryseobacterium indologenes*, and 2. IA132 were also grown to adulthood on OP50-1 and were pre-cleared of OP50-1 prior to exposure to *C. nematophagum*. Following a 48-h exposure of IA132 to *C. indologenes*, there was no degradation of multimeric tagged collagen COL-12 (Fig. 4 lane 4) and this is in contrast to the 48-h exposure of the OP50-1 pre-cleared adults to either JUb129 or JUb275 (Fig. 5, lanes 5 and 6) which completely degraded the tagged collagen.

### Parasitic nematode killing

As this bacterium was found to infect and kill bacterivorous nematodes, we tested it for killing activity against a number of field and laboratory isolates of parasitic nematodes including the significant Trichostrongylid and Strongylid pathogens of livestock and domesticated animals. The list of nematodes species tested is presented in Table 2 and includes animal parasites of sheep, cattle, horses, opossums, rats, wolves and a plant parasite of potatoes. All nematode stages and species were tested in a similar manner to *C. elegans*, namely, eggs or larvae were place on fresh bacterial lawns of *C. nematophagum* on NGM plates and compared to those placed on OP50-1. All free-living (L1–L3) bacterivorous stages of all nematodes tested were infected and killed by *C. nematophagum* in a similar manner as described from *C. elegans*, whereas no death was noted on OP50-1 culture. Killing rates were quantified for the L1 but not the L2 or L3 stages. Upon ingestion of bacteria, the L1 larvae became immotile and 100% larvae are dead at 24 h (Table 2). Major pathology involves infection and digestion of the pharynx followed by rupture into the body cavity and internal digestion of the nematode (Fig. 5 and Additional file 6). The infection and multiplication within the L2 larvae of *Haemonchus contortus*, the important Trichostrongylid gastrointestinal parasite of sheep, is shown in Fig. 5. *H. contortus* eggs hatch and develop from L1 to L3 stage on OP50-1 seeded NGM plates (Fig. 5a), whereas exposure of the L2 stage to *C. nematophagum* on NGM plates results in destruction of the anterior pharynx (Fig. 5b and c) and eventual filling of the body cavity with bacilli (Fig. 5d). Similar infections are depicted in a wide range of Strongylid and Trichostrongylid parasites (Additional file 6). The only species tested that was not killed was the potato parasitic nematode *Globodera pallida* (Additional file 6 L), and this probably reflects the non-bacterial diet and the presence of mouthparts that are specialised for piercing and feeding on plant roots. In addition, we tested this bacterial species against the larval stages of insects, namely *Aedes aegypti* mosquitos and no killing or pathology was noted (Additional file 7).

**Table 2:** Nematode species killed by *Chryseobacterium nematophagum*

Nematode species (host)	Killing	Stages killed	% L1 killed in 24 h (number counted)*
*Caenorhabditis elegans* (free-living)	+	All stages	100% (0/193)
*Caenorhabditis briggsae* (free-living)	+	All stages	Not determined
*Globodera pallida* (potato) J2 and J3	–	None	Not determined
*Haemonchus contortus* ISE and IRE strains (sheep and goats)	+	L1, L2 and L3	100% (IRE 0/215)
*Trichostrongylus vitrinus* (sheep and goats)	+	L1, L2 and L3	100% (0/56)
*Teladorsagia circumcincta* (sheep and goats)	+	L1, L2 and L3	100% (0/201)
*Cyathastomin* sp*.* (horses)	+	L1, L2 and L3	100% (0/143)
*Ostertagia ostertagi* (cattle)	+	L1, L2 and L3	100% (0/158)
*Parastrongyloides trichosura* (opossum)	+	All free-living stages	Not determined
*Cooperia curtecei* (sheep and goats)	+	L1, L2 and L3	100% (0/47)
*Cooperia oncophera* (cattle)	+	L1, L2 and L3	100% (0/54)
*Nippostrongylus brasiliensis* (rats and mice)	+	L1, L2 and L3	100% (0/314)
*Ancylostoma caninum* (dogs, wolves and foxes)	+	L1, L2 and L3	100% (0/44)

*A 24-h survival rate relates to the number of freshly hatched L1s larvae added to NGM plates seeded with *Chryseobacterium nematophagum* that have survived after a 24-h culture period (number surviving 24 h/number of L1 added)

### Comparative genomics

**Fig. 6:**
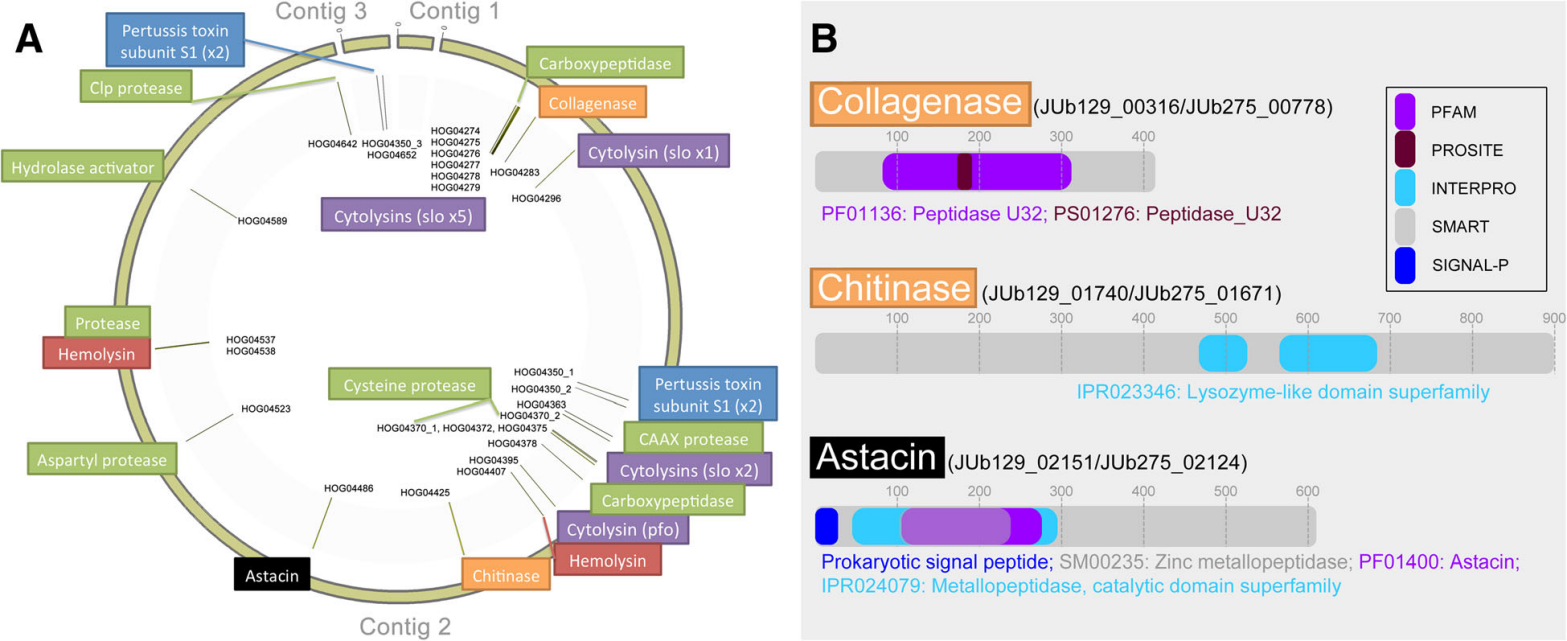
Genomic loci of candidate genes and domain structure of collagenase, chitinase and astacin enzymes*.* **a** Genomic location of nematode killing phenotype-associated candidate genes as identified in the top-ranking hierarchical orthologous groups. This excludes the Por gene family, which is illustrated in Additional file 12. **b** The domain architecture of the *C. nematophagum*-specific collagenase, chitinase and astacin proteins is illustrated with the IDs for the orthologous sequences in JUb129 and JUb275 shown. The scale represents the number of amino acid residues, and the legend shows the relevant protein motif database

The genomes of JUb129 and JUb275 were predicted to encode 3738 and 3586 protein sequences, respectively. Annotated genomic sequence files are provided as Additional files 8 and 9. In order to investigate which genes might be involved in conferring the nematode-killing ability of *C. nematophagum*, the two genomic sequences representing this species were compared to that of five other *Chryseobacterium* spp. known not to possess the nematode-killing phenotype. A total of 5020 sets of orthologous genes were identified, which were organised into 4657 hierarchical orthologous groups (HOGs), detailed in Additional file 10. Only 77 HOGs represented in JUb275 were not detected in JUb129 (1.66%), while only 136 HOGs represented in JUb129 were not detected in JUb275 (2.92%), illustrating the high degree of similarity between these two annotated assemblies. The entire set of HOGs was screened to identify which ones were specific to or expanded within *C. nematophagum*. Three hundred eighty-two such HOGs were identified (Additional file 11), representing about 13% of the *C. nematophagum* genome, the majority of which were identified as *C. nematophagum*-specific. The ability to digest the nematode cuticle and the pharyngeal lining are unusual properties for a bacterium and predicted to be carried out by specific collagenases and chitinases. In order to identify these together with other genes of interest, a further subset of HOGs was identified where gene annotation contained terms such as ‘protease’, ‘peptidase’, ‘collagenase’, ‘chitinase’, ‘gelatinase’, ‘lysin’ or ‘toxin’. This resulted in the identification of 24 high-value candidate HOGs (Table 3). The genomic locations of these genes are illustrated in Fig. 6a and Additional file 12. These candidate genes include *C. nematophagum*-specific collagenase, chitinase and astacin encoding genes; the primary domain structure of these three key enzymes is illustrated in Fig. 6b. The collagenase enzyme is a 414-amino acid protein that is completely conserved at the amino acid level between the two *C. nematophagum* isolates. The chitinase is an 899-amino acid protein that has 93% identity while astacin comprises 610/614 amino acid residues, sharing 92% identity between isolates. The astacin protein contains an N-terminal prokaryotic secretion signal indicated it is secreted across the inner membrane.

**Table 3:** Nematode-killing candidate genes

Hierarchical orthologous group (HOG)	Annotation	Number of copies		PFAM domain
		JUb 129	JUb 275	
HOG04283	Collagenase	1	1	PF01136
HOG04425	Chitinase	1	1	–
HOG04486	Flavastacin precursor, Astacin	1	1	PF01400
HOG04350	Pertussis toxin subunit S1	3	1	–
HOG04652	Pertussis toxin subunit S1	1	1	–
HOG04395	Thiol-activated cytolysin (pfo)	1	1	PF01289
HOG04296	Thiol-activated cytolysin (slo1)	1	1	PF01289
HOG04279	Thiol-activated cytolysin (slo2)	1	1	PF01289
HOG04278	Thiol-activated cytolysin (slo3)	1	1	PF01289
HOG04277	Thiol-activated cytolysin (slo4)	1	1	PF01289
HOG04276	Thiol-activated cytolysin (slo5)	1	1	PF01289
HOG04275	Thiol-activated cytolysin (slo6)	1	1	PF01289
HOG04372	Thiol-activated cytolysin (slo7)	1	1	PF01289
HOG04375	Thiol-activated cytolysin (slo8)	1	1	PF01289
HOG04370	Cysteine protease, C1A family^a^	2	2	PF00112
HOG04523	Retroviral aspartyl protease	1	1	PF00077
HOG04537	Protease/peptidase	1	1	PF00664
HOG04274	d-alanyl-d-alanine carboxypeptidase precursor	1	1	PF00144
HOG04378	d-alanine carboxypeptidase	1	1	PF00144
HOG04589	nlpD Murein hydrolase activator	1	1	PF01551
HOG04363	CAAX amino terminal protease	1	1	PF02517
HOG04642	ATP-dependent Clp protease	1	1	PF00004
HOG04407	Hemolysin	1	1	PF12700
HOG04538	Hemolysin	1	1	–
24 × HOGs	Por gene^b^	38	38	PF00041; PF07593; PF11958; PF04231; PF01421;PF02128

^a^Single copy present in *C. shigense* genome

^b^Also present in other *Chryseobacterium* spp. genomes: *C. contaminans* (*n* = 8), *C. gallinarum* (*n* = 7), *C. indologenes* (*n* = 6), *C. indoltheticum* (*n* = 4) and *C. shigense* (*n* = 6)

Interestingly, the *C. nematophagum* genome contains two Pertussis toxin S1 subunit-encoding genes, one of which has expanded to three paralogues in JUb129. The presence of these toxin-encoding genes was assessed across all 120 *Chryseobacterium* spp. genomes currently available in the NCBI database, and these were found to be absent, indicating they are likely the result of a lateral gene transfer event in the recent evolutionary history of this species. Strikingly, and also specific to the *C. nematophagum* genome, nine members of a thiol-activated cytolysin family, the *slo* genes, were identified.

Analysis of the genome of *C. nematophagum* has allowed us to identify all the major components of the gliding and PorS/Type IX secretion system genes; with orthologues of GldA, B, C, D, E, F, G, H, J, K, L, M, N and SprT and SprA all being present. Interestingly, the PorS genes GldK, GldL, GldM and GldN are found clustered in a single region in the genome (Additional file 12), perhaps representing an operon. This secretion system is known to be associated with virulence and gliding motility in the phylum Bacteroidetes, of which *Chryseobacterium* is a member.

## Discussion

In this study, we have identified and characterised a novel environmental *Chryseobacterium* species with potent nematocidal properties that we have named *Chryseobacterium nematophagum*. Two separate, but very closely related isolates, were isolated from Europe (Paris) and Asia (Bangalore), both were found associated with and colonising the free-living nematode *Caenorhabditis briggsae*, and both isolates of infected nematodes were found associated with rotting fruit and the accompanying bacterial flora. *Chryseobacterium nematophagum* rapidly kills both *C. briggsae* and the sister species *C. elegans*, but more significantly, this *Chryseobacterium* infects and kills all bacterivorous stages of all the parasitic nematodes tested to date, indicating that if ingested, these bacteria will kill and colonise many nematode species. Nematode killing was rapid, occurring within 3 h of ingestion and involved the digestion of the anterior pharyngeal lining followed by bacteria invasion and colonisation of the body cavity. All nematode tissues were ultimately consumed, including the normally highly insoluble cuticular exoskeleton. Both the pharynx and the cuticle are composed of large highly crosslink non-reducible collagens [21, 22] and complex carbohydrate macromolecules such as chitin [24].

Digestion of these matrix materials requires the action of active collagenase and chitinase enzymes, members of which are uniquely encoded in the genome of the pathogenic bacteria analysed in this study. Nevertheless, it is interesting to note that chitinase, gelatinase and collagenase metalloprotease activities have all been described in related *Chryseobacterium* species and have been linked with gliding motility, PorS type IX secretory systems and virulence characteristics [25]. It is also significant to note that these bacteria have neither collagen nor chitin proteins or structures. *Chryseobacterium* species belong to the Bacteriodetes phylum, members of which are being increasingly describes as having unusually linked motility (gliding, Gld) and secretory system (PorS, Spr) [25, 26]. It is also significant to note that one of the two chitinases also possess a C-terminal PorS domain, indicating that this enzyme should be secreted from this bacterium. This PorS type IX secretory system differs from the well-defined type I–VI bacterial secretory systems and differs from the mycobacterial type VII system and the type VIII systems [26]. The *C. nematophagum* gliding and PorS components are very similar to those of other Bacteroidetes such as *Flavobacterium johnsoniae* [25]. The importance of these PorS-secreted digesting enzymes is clearly demonstrated in *Chryseobacterium* sp. strain kr6, which was isolated from poultry industry waste and found to degrade chicken feathers via the action of a specific keratinase enzyme [12].

Analysis of the *C. nematophagum* genome has identified genes that encode additional PorS-secreted proteins that may be involved in matrix digestion and virulence, with the secreted astacin metalloproteases, chitinases and collagenases being particularly good candidates for future characterisation with respect to virulence.

By applying a *C. elegans* transgenic collagen reporter strain, we have also demonstrated by Western blotting that this bacterium can digest the highly insoluble cross-linked cuticle collagens (Fig. 4). Additionally, by analysing an mCherry pharyngeal transgenic marker strain and a chitosan-specific stain, we can observe the physical destruction of this collagen and chitin-lined structure which occurs in less than 3 h (Fig. 3).

## Conclusions

We have investigated the ability of *C. nematophagum* to kill and digest the environmental stages of field isolates of important Trichostrongyle and Strongyle nematodes of livestock and domesticated animals. Invasion and digestion proceeds in a similar fashion to that described for the model nematode *C. elegans*. Most notably, *C. nematophagum* kills the environmental L1–L3 stages of an anthelmintic resistant strain (IRE) of the sheep parasite *Haemonchus contortus*. This bacterium is also highly effective against the L1–L3 stages of the horse Cyathostomins, a group of Strongyle nematodes that are becoming increasingly resistant to all the available anthelmintic classes. This pathogenicity raises the possibility that *C. nematophagum*, or indeed its isolated virulence factors, could provide a future novel means of controlling these increasingly problematic parasites of grazing livestock. Ultimately, it may also provide an alternative control measure to fight the pathogenic soil-transmitted helminths of humans, including, for example, the important hookworm parasites, *Ancylostoma duodenale* and *Necator americanus*, both of which are related to the wolf hookworm *Ancylostoma caninum* and rat hookworm *N. brasilliensis*, which are both highly susceptible to killing by this bacterium. We find that *C. nematophagum* grows efficiently in the presence of nematodes and that *C. elegans* are attracted to and not repelled by this bacterium, suggesting that this represents a true host/pathogen interaction.

## Methods

### Nematode strains, culture and killing assays

*C. elegans* N2, VS21 [*myo-2p::mCherry*] and *C. briggsae* (AF16) strains were supplied by the *C. elegans* Genetics Centre (CGC). The TY-tagged col-12 strain IA132 was provided by Iain Johnstone (University of Glasgow). All *Caenorhabditis* strains were maintained on NGM agar plates supplemented with *E. coli* bacteria OP50-1 following standard techniques http://www.wormbook.org/toc_wormmethods.html.

The lab-derived drug sensitive *Haemonchus contortus* strain MHco3(ISE) and drug-resistant MHco18(IRE), and field isolates of the following *Trichostrongylids: H. contortus*, *Trichostrongylus vitrinus*, *Teladorsagia circumcincta*, *Ostertagia ostertagi*, *Cooperia curticei* and *Cooperia oncophera* were all kindly provided by Dave Bartley and Alison Morrison from the Moredun Research Institute. Additional field isolates of *T. circumcincta* were provided by George King (University of Glasgow), and the *Cyathastomin* parasites were provided by Ronnie Barron (University of Glasgow). *Globodera pallida* J2 larvae were provided by Aaron Maule (Queens University), *Nippostrongylus brasilliensis* was proved by Rick Maizels (University of Glasgow), *Ancylostoma caninum* was provided by Elizabeth Schmidt (Botucatu, Brazil) and *Parastrongyloides trichosura* was provided by Adrian Streit (Max-Planck, Tuebingen). For the Trichostrongylids and Strongylids, embryos were purified from faecal samples derived from either mono-specifically infected donor lambs or naturally infected ruminants, via saturated salt flotation. The eggs were hatched to L1 and developed to L2 and L3 by culturing on NGM agar OP50-1 as per *C. elegans*. Free-living *P. trichosura* and *Globodera pallida* J2 larvae were cultured on NGM plates as per *C. elegans*.

### Nematode killing assays

All nematode stages and species were tested by placing either 100 freshly prepared eggs or 50–100 L1 larvae on fresh bacterial lawns (200 μl of overnight culture in SOB media) of *C. nematophagum* on 5 cm NGM plates, and survival and morphology was observed over 2 to 3 days and compared to those placed on OP50-1. Time-course experiments were carried out on L1 larvae derived from bleach-treated hermaphrodite egg preparation (10 min, 250 mM KOH/1% bleach). Eggs were washed and allowed to hatch overnight in the absence of bacteria, then L1s were added to bacterial lawns on NGM plates. To obtain synchronised L2, L3 and L4s, the larvae were collected at various time-points from OP50-1 fed L1s. Likewise, the pharyngeal labelled strain, VS21, was bleach treated to derive eggs then L1s that were observed for pharyngeal damage via U.V. microscopic analysis.

### Chitosan staining

The chitosan staining protocol followed a modified version of a previously published method [20]. Briefly, freshly prepared L1s of *C. elegans* were washed and then suspended in 500 μl citrate-phosphate buffer, pH 6 (0.2M NaH_2_PO_4_ and 0.1M K citrate) prior to adding 15 μl eosin *Y* stock (5 mg/ml in 70% ethanol). Tubes were incubated in the dark for 10 min, then washed extensively in citrate phosphate buffer before adding to *C. nematophagum*-inoculated NGM plates. Samples were observed, and images collected over a 6-h period.

### Imaging and microscopy

All nematodes were either viewed on plates using a Zeiss bench-top microscope fitted with a Canon Sureshot camera or were mounted on 2% agar pads on slides and viewed under Differential Interference Contrast (DIC) or fluorescence optics on a Zeiss Axioscop2 and imaged with a Zeiss AxioCam camera and Axiovision software.

### DNA extraction and whole-genome sequencing

Overnight cultures of JUb129 and JUb275 each in 10 ml SOB were re-suspended in 2 ml 25% sucrose in TE, digested in 100 mg/ml Roche lysozyme followed by 20 mg/ml proteinase K digestion. Ten milligrams per milliliter of RNAse was added prior to adding 400 μl 0.5M EDTA and 30 μl of 10% sarcosyl and leaving at 50 °C overnight. Samples were made up to 12 ml with TE prior to phenol/chloroform extraction and ethanol precipitation. Samples were then sent to Wellcome Trust Sanger Institute for whole genome sequencing using the PacBio platform.

For JUb129, PacBio RS sequence reads were assembled using HGAP v3 [27] of the SMRT analysis software package v2.3.0. The fold coverage was set to 30, and the approximate genome size was set to 3 Mb. The assembly was circularised using Circlator v1.1.3 [28] and polished using the PacBio RS resequencing protocol and Quiver v1 [27] of the SMRT analysis software package v2.3.0. For JUb275, PacBio RSII reads were converted to BAM format using the SMRTlink pipeline v5.0.1.9585 and then to FASTQ format using Samtools v1.6 [29], excluding reads which failed quality control. Reads were assembled using CANU v1.6 [30]. The assembly was circularised using Circlator v1.5.3 [28]. The PacBio SMRTlink resequencing pipeline was run utilising Quiver [27], and the corrected reads mapped back to the final assembly using minimap2 v2.6 [31]. For both genomes, automated annotation was undertaken using PROKKA v1.11 [32] based on a genus-specific NCBI Reference Sequence (RefSeq) database. Protein motifs were identified on the basis of matching domains in the UniProtKB [33], TIGRFAM [34], PFAM [35] and NCBI protein cluster [36] databases. Genomic sequence data has been deposited in the NCBI database under BioProject number PRJNA487926 and BioSamples numbered SAMN09925763 (JUb129) and SAMN09925764 (JUb275). Annotated genomic sequence data is available in Additional file 8 (JUb129) and Additional file 9 (JUb275).

### Phylogenetics

A maximum likelihood tree, based on the 16S SSU rRNA gene, was generated using RAXML [37] using a generalised time-reversible model of sequence evolution. The tree was constructed using the JUb129 and JUb275 sequences together with a representative collection of *Chryseobacterium* spp. sequences downloaded from the NCBI database, including that of *C. lactis (LN995695.1)*, *C. indologenes (*JX515610.1), *C. jejuense (JX035956.1)*, *C. shigense (NR_041252.1)*, *C. oleae (NR_134002.1)*, *C. contaminans (NR_133725.1)*, *C. gallinarum (*CP009928.1), *C. gleum (*FJ887959.1), *C. joostei (KU058436.1)*, *C. shandongense (NR_135879.1)*, *C. daecheongense (*KJ147083.1), *C. hispalense (NR_116277.1)*, *C. daeguense (NR_044069.1)*, *C. polytrichastri (NR_134710.1)*, *C. indoltheticum (NR_042926.1)*, *C. pallidum* (NR_042504.1) and *C. taichungense (*JX042458.1). The 16S sequence of another member of the Flavobacteriaceae family, *Riemerella anatipestifer* (CP006649.1_2), was used as an out-species to root the tree. Stability was assessed using 100 bootstrap pseudo-replicates, and the tree was visualised using FigTree 1.4 (http://tree.bio.ed.ac.uk).

### Comparative genomics

Orthologous genes were defined across the genomes of JUb129, JUb275 along with five *Chryseobacterium* spp. known to not possess the nematode-killing phenotype, namely *C. contaminans* (GCA_001684955.1), *C. gallinarum* (GCA_001021975.1), *C. indologenes* (GCF_001295265.1), *C. indoltheticum* (GCA_900156145.1) and *C. shigense* (GCA_900156575.1). Annotated genomic sequence data for these species was downloaded from the NCBI Genome database. Pairs of orthologous sequences were identified using a stand-alone version of the Orthologous Matrix (OMA) algorithm v2 [38], following which hierarchical orthologous groups were inferred [39] (Additional file 11). Top-ranking ‘nematode-killing’ candidate genes were analysed for known amino acid motifs using InterProScan [40]. These sequences were then compared to the 120 *Chryseobacterium* spp. genomes currently available in the NCBI Genome database (Additional file 13) using the BLASTP 2.2.26+ [41].

### Analysis of collagenase activity of *C. nematophagum*

Twenty young adults of the COL-12 TY tagged *C. elegans* strain (IA132) were picked from OP50-1 NGM plates and incubated in microtitre wells containing 100 μl M9 buffer and 10 μl *C. nematophagum* broth for 24–48 h with controls being identically treated IA132 with OP50-1 or *Chryseobacterium indologenes* for 48 h. To exclude the involvement of OP50-1 in collagen digestion, a set of experiments included adult IA132 worms grown on OP50-1 which were washed three times in M9 and cultured for 4 h on unseeded NGM plates prior to culturing with JUb129 and JUb275 for 48 h. All wells were setup in triplicate and well contents were transferred to Eppendorfs and centrifuged 1000 rcf for 2 min and the pellets frozen at − 20 °C. Pelleted worms were resuspended in 1× SDS PAGE sample buffer with 5% mercaptoethanol and boiled for 10 min, centrifuged and supernatant added to wells of 4–20% mini-protean Bio-Rad SDS PAGE gels and run at 200 v for 30 min. Western blotting was carried out on a Bio-Rad Mini Transfer Cell following the manufacturer’s recommendations. The PVDF membrane (GE Healthcare) was removed, blocked in 5% marvel PBS 0.1% tween, probed with anti-TY tag and then goat anti-mouse HRP, and this was followed by detection with Pierce ECL plus substrate. Blots were then stripped and re-probed with anti-actin antibody.

### Microbiology

Analytical Profile Index (API) strips (20E and 29NE) were analysed following the manufacturer’s instruction (Biomerieux) by incubating at 30 °C for 24 and 48 h. Oxidase tests were carried out by selecting a colony of *C. nematophagum* on a cotton bud and adding drops of tetramethyl-*p*-phenylenediamine dihyrochloride. Gram staining was performed on a slide spread of *C. nematophagum* following conventional methods [42]. Flexirubin tests were performed on *C. nematophagum* colonies using 20% KOH as described [43].

## Additional files

Additional file 1: Bacteriological characterisation of *Chryseobacterium nematophagum* JUb129 and JUb275: growth on 5% sheep blood plates, flexirubin test and gram stain (PDF 3681 kb)

Additional file 2: Bacteriological characterisation: API results for *Chryseobacterium nematophagum* JUb129 (PDF 393 kb)

Additional file 3: Larval survival assays: survival of *Caenorhabditis elegans* L1, L2, L3 and L4 on *Chryseobacterium nematophagum* lawns. (PDF 311 kb)

Additional file 4: Concentration and ratio of *Chryseobacterium nematophagum* (JUb275) to *Escherichia coli* (OP50-1) required to kill *Caenorhabditis elegans elegans* larvae after 24 h exposure. (PDF 158 kb)

Additional file 5: *Caenorhabditis elegans* attraction assays to *Chryseobacterium nematophagum*. (PDF 144 kb)

Additional file 6: Killing of larval parasitic nematode species on exposure to *Chryseobacterium nematophagum*. (PDF 9871 kb)

Additional file 7: Testing of *Chryseobacterium nematophagum* on mosquito larvae. (PDF 104 kb)

Additional file 8: Annotated *Chryseobacterium nematophagum* isolate JUb129 genome sequence. (GFF 5850 kb)

Additional file 9: Annotated *Chryseobacterium nematophagum* isolate JUb275 genome sequence. (GFF 5585 kb)

Additional file 10: Hierarchical orthologous genes defined across *Chryseobacterium* spp. with full genomic annotation. (XLSX 1271 kb)

Additional file 11: List of 382 top-ranking hierarchical orthologous candidate genes specific to or expanded within *Chryseobacterium nematophagum.* (XLSX 124 kb)

Additional file 12: Distribution of PorS genes in the *Chryseobacterium nematophagum* genome. (PDF 401 kb)

Additional file 13: *Chryseobacterium* spp. genomes available in NCBI Genome database. (PDF 54 kb)
