# Supplementary material for: The golden death bacillus Chryseobacterium nematophagum is a novel matrix digesting pathogen of nematodes
Source: BMC Biol. 2019 Feb 28;17:10. doi: 10.1186/s12915-019-0632-x (PMC6394051; doi:10.1186/s12915-019-0632-x)
Supplement: Supplementary file 1 — Bacteriological characterisation of Chryseobacterium nematophagum JUb129 and JUb275: growth on 5% sheep blood plates, flexirubin test and gram stain (PDF 3681 kb) [file 12915_2019_632_MOESM1_ESM.pdf]

Bacteriological characterisation of JUb129 and JUb275

A.

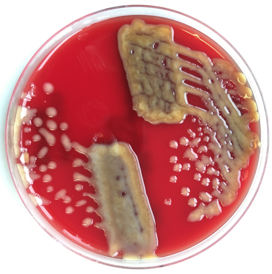

B.

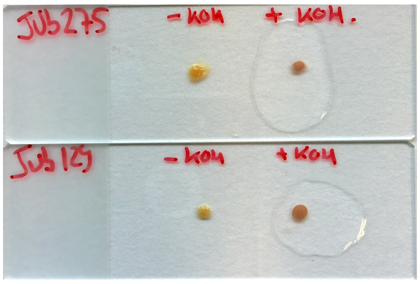

C.

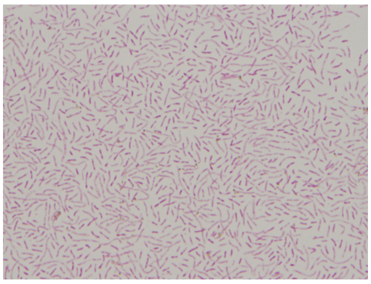

A. JUb275 yellow colonies grow in 5% sheep blood agar; **B.** Flexirubin test on colonies of JUb275 (top) and JUb129 (bottom), reveals red colour change in presence of 20% KOH; **C.** Pink gram negative stain on JUb129.
