# Supplementary material for: The golden death bacillus Chryseobacterium nematophagum is a novel matrix digesting pathogen of nematodes
Source: BMC Biol. 2019 Feb 28;17:10. doi: 10.1186/s12915-019-0632-x (PMC6394051; doi:10.1186/s12915-019-0632-x)
Supplement: Supplementary file 2 — Bacteriological characterisation: API results for Chryseobacterium nematophagum JUb129 (PDF 393 kb) [file 12915_2019_632_MOESM2_ESM.pdf]

API results for JUb129

**20E API test**	**20E results**	**20NE API test**	**20NE results**
o-nitrophenyl-β-D-galactopyranoside (ONPG)	Negative	Potassium nitrate (NO_3)	Negative
Arginine dihydrolase (ADH)	Negative	L-tryptophane (TRP)	Negative
Lysine decarboxylase (LDC)	Negative	D-glucose (GLU)	Negative
Ornithine decarboxylase (ODC)	Negative	L-arginine (ADH)	Negative
Citrate (CIT)	Negative	Urea (URE)	Negative
Hydrogen sulphide (H_2S)	Negative	**Esculin (ESC)**	**Positive**
Urease (URE)	Negative	**Gelatin (GEL)**	**Positive**
Tryptophan deaminase (TDA)	Negative	4-nitrophenyl-βDgalactopyranoside (PNG)	Negative
Indole (IND)	Negative	D-glucose (GLU)	Negative
Voges-proskauer (VP)	Negative	L-arabinose(ARA)	Negative
**Gelatin (GEL)**	**Positive**	D-mannose(MNE)	Negative
Glucose (GLU)	Negative	D-mannitol (MAN)	Negative
Mannose (MAN)	Negative	**N-acetyl-glucosamine (NAG)**	**Positive**
Inositol (INO)	Negative	D-maltose (MAL)	Negative
Sorbital (SOR)	Negative	Potassium gluconate (GNT)	Negative
Rhamnose (RHA)	Negative	Capric acid (CAP)	Negative
Sucrose (SAC)	Negative	Adipic acid (ADI)	Negative
Melibiose (MEL)	Negative	Malic acid (MLT)	Negative
Amygdalin (AMY)	Negative	Trisodium citrate (CIT)	Negative
Arabinose (ARA)	Negative	Phenylacetic acid (PAC)	Negative

JUb129 results for each biochemical test in the 20E and 20NE API strips
