# Supplementary material for: The golden death bacillus Chryseobacterium nematophagum is a novel matrix digesting pathogen of nematodes
Source: BMC Biol. 2019 Feb 28;17:10. doi: 10.1186/s12915-019-0632-x (PMC6394051; doi:10.1186/s12915-019-0632-x)

Larval Survival Assays

Caenorhabditis elegans gravid hermaphrodites were bleach treated then allowed to hatch overnight in M9 buffer minus bacteria. Synchronised L1 larvae were then added to plates and observed for the L1 survival studies. To obtain L2 larvae, the synchronised L1 larvae were cultured on OP50 plates for 24 hours at 16 °C, for L3 larvae synchronised L1 larvae were cultured on OP50 plates for 24 hours at 20 °C and to obtain L4 larvae, synchronised L1 larvae were cultured for 27 hours at 25 °C. Following visual checking by microscopy, the semi-synchronous L2, L3 and L4 larvae were washed free from OP50 in M9 buffer and placed on bacteria free NGM plates for one hour prior to washing in M9 buffer and then placing on the experimental plates for survival analysis and counting. Multiple NGM plates with 100 µl of bacteria (OP50 or JUb275) were inoculated with approximately 100 larvae per plate and survival was assessed microscopically over a 24hour period.

Table. Survival of *C. elegans* larval stages on *C. nematophagum* lawns

	**L1 stage**	**L1 stage**	**L2 stage**	**L3 stage**	**L4 stage**
Time (hrs)	OP50 % alive (n=241)	JUb275 % alive (n=193)	JUb275 % alive (n=395)	JUb275 % alive (n=372)	JUb275 % alive (n=163)
0	100	100	100	100	100
1	100	97.4	54	86	90
2	99.5	88.6	38	43	73
3	99.5	64.7			55
4	99.2	44	32	11	28
5	98.3	32			23
6	98.3	23.3			
7	98.3	0			
24	97.2	0	0	0	0

Figure. Plate images (x400) of A. *C. elegans* N2 L2 on OP50 after 24 hr exposure and B, Dead *C. elegans* N2 L2 after 24 hr exposure to JUb275 culture.

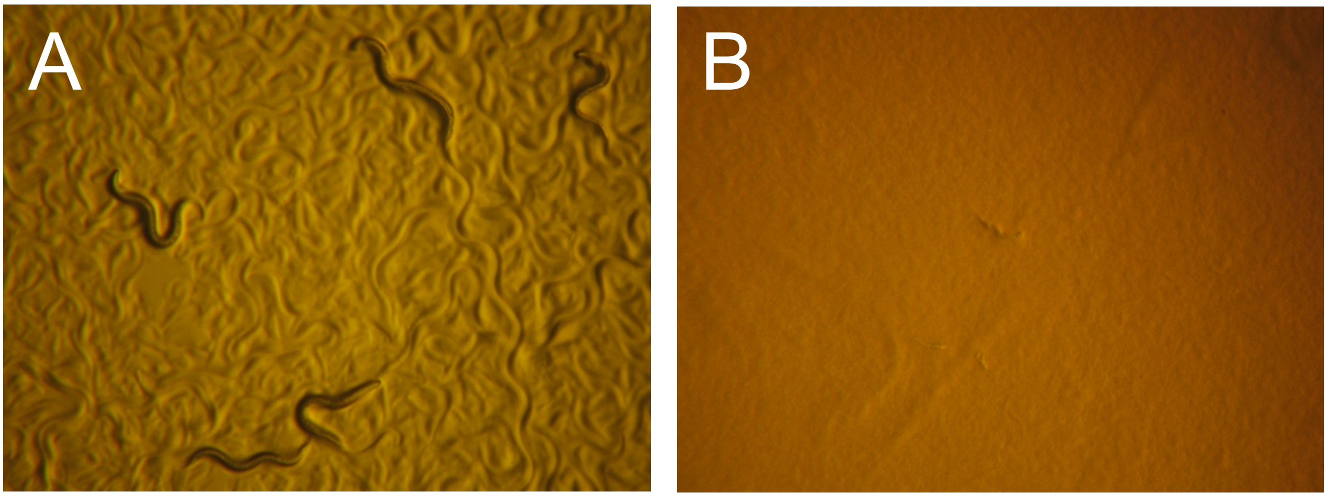

Supplement: Supplementary file 3 — Larval survival assays: survival of Caenorhabditis elegans L1, L2, L3 and L4 on Chryseobacterium nematophagum lawns. (PDF 311 kb) [file 12915_2019_632_MOESM3_ESM.pdf]
