# Supplementary material for: The golden death bacillus Chryseobacterium nematophagum is a novel matrix digesting pathogen of nematodes
Source: BMC Biol. 2019 Feb 28;17:10. doi: 10.1186/s12915-019-0632-x (PMC6394051; doi:10.1186/s12915-019-0632-x)

Concentration and ratio of *C. nematophagum* (JUb275) to *E. coli* (OP50-1) required to kill *C. elegans* larvae after 24 hour exposure

Set up overnight cultures of JUb275 (30 °C) and OP50-1 (37 °C) in SOB media from single colonies. Measured OD600 and calculated colony forming units per dilution following serial dilutions and plating and counting colonies following overnight culture on 5% sheep blood plates.

**Bacterial counts**	**cfu/µl**
JUb275	2.24×10^{6}
OP50-1	2.99×10^{6}

To a set of duplicate or triplicate 55cm NGM agar plates, the following broth (200 µl) were set up and then 50 freshly prepared *C. elegans* per plate were added and viability was scored after 24 hours.

Plate	No. L1s	JUb275 *cfu*	OP50-1 *cfu*	Ratio	L2 surviving 24 hrs (%)
**10**	153	4.48×10^{8}	0	-	0
**9**	164	0	5.98×10^{8}	-	158 (96%)
**8**	98	2.24×10^{8}	5.98×10^{8}	7.50×10^{-1}	0
**7**	100	1.79×10^{8}	2.99×10^{8}	5.00×10^{-1}	0
**6**	106	1.34×10^{8}	3.58×10^{8}	3.21×10^{-1}	0
**5**	90	8.96×10^{7}	4.18×10^{8}	1.88×10^{-1}	0
**4**	112	4.48×10^{7}	4.78×10^{8}	8.33×10^{-2}	0
**3**	72	2.24×10^{7}	5.38×10^{8}	3.95×10^{-2}	0
**2**	135	1.12×10^{7}	5.68×10^{8}	1.92×10^{-2}	0
**1**	138	2.24×10^{6}	5.82×10^{8}	3.77×10^{-3}	0
**14**	86	2.24×10^{5}	5.94×10^{8}	3.77×10^{-4}	0
**15**	112	4.48×10^{4}	5.94×10^{8}	7.54×10^{-5}	0
**11**	105	2.24×10^{4}	5.94×10^{8}	3.77×10^{-5}	0
**12**	99	**2.24×10^{2}**	**5.94×10^{8}**	**3.80×10^{-7}**	**0**
**13**	110	**2.24**	**5.94×10^{8}**	**3.77×10^{-9}**	**99 (90%)**

Therefore 200 *cfu* of *C. nematophagum* diluted in 38,000,000 OP50-1 will kill 100% of L1 larvae after 24 hour exposure.

Representative 55mm Plate images: golden colonies of JUb275 on transparent lawn of OP50-1

12. 200 *cfu* JUb275

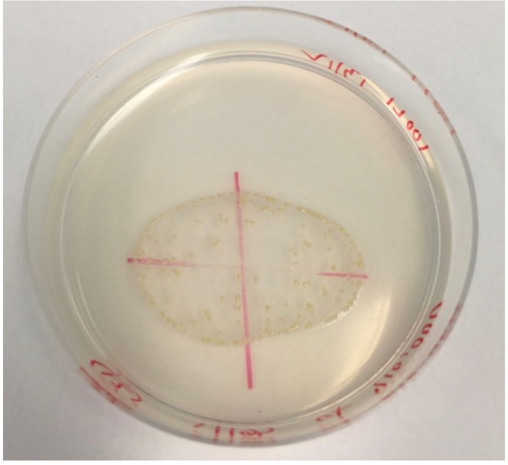

13. 1 *cfu* JUb275

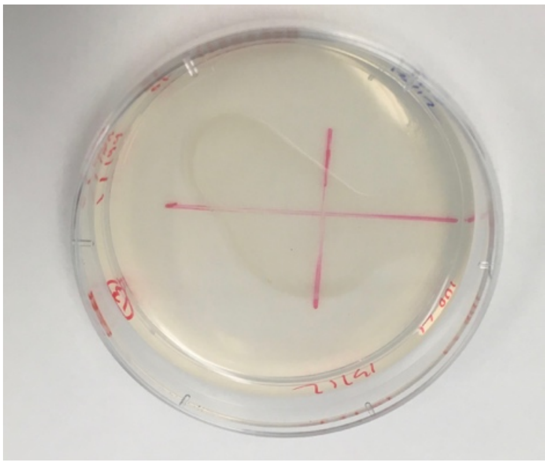

Supplement: Supplementary file 4 — Concentration and ratio of Chryseobacterium nematophagum (JUb275) to Escherichia coli (OP50-1) required to kill Caenorhabditis elegans elegans larvae after 24 h exposure. (PDF 158 kb) [file 12915_2019_632_MOESM4_ESM.pdf]
