# Supplementary material for: The golden death bacillus Chryseobacterium nematophagum is a novel matrix digesting pathogen of nematodes
Source: BMC Biol. 2019 Feb 28;17:10. doi: 10.1186/s12915-019-0632-x (PMC6394051; doi:10.1186/s12915-019-0632-x)
Supplement: Supplementary file 5 — Caenorhabditis elegans attraction assays to Chryseobacterium nematophagum. (PDF 144 kb) [file 12915_2019_632_MOESM5_ESM.pdf]

Caenorhabditis elegans attraction assays to *Chryseobacterium nematophagum*

Wild type (N2) *C. elegans* hermaphrodites were bleach treated to purify embryos which were hatched overnight in M9 buffer in the absence of food. Approximately 50 synchronised L1 larvae were added to Point X of 55 mm NGM agar plates that were seeded with 25 µl of overnight culture of bacteria. After 24 hours at 22 °C, larvae from 4 replicates were counted in the bacterial lawn at circled position O and outside the lawn for both OP50-1 and JUb275 seeded plates.

Figure. Bacterial attraction plate assay

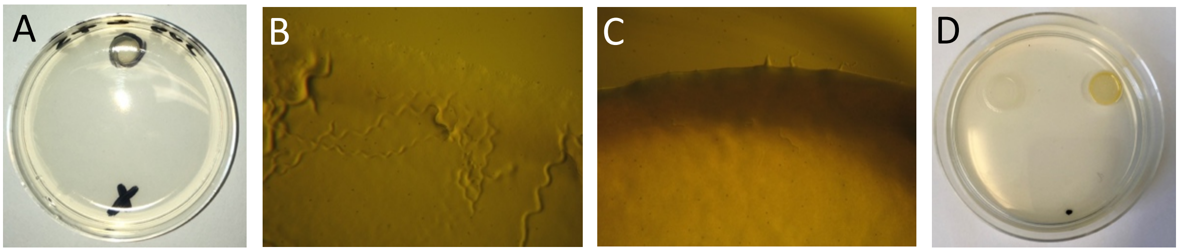

A. L1s added to X on 55 mm NGM agar plate and position relative to lawn (circled) noted after 24 hours, **B.** active L2 and L3 larvae noted on OP50 lawn x160, **C.** Dead and decaying L1 larvae noted on JUb275 plate, **D.** L1 migration preference on single plate; 400 L1s added to single point (black spot), and position relative to JUb275 (right golden lawn) and OP50-1 lawn (left transparent lawn) noted after 24 hours.

Nematodes attracted to bacterial lawn after 24 hours

Bacteria	Total N2 on 4 plates	Number off lawn	Number on Lawn	% attracted to Lawn
OP50-1	192	66	120	62.5%
JUb275	197	74	123	62.4%

Next a single plate was set up with two opposing spots (D above), 25 µl of overnight JUb275 culture (golden lawn, right) and 25 µl OP50-1 overnight culture (transparent lawn, left). 400 freshly prepared L1 larvae were added to the black spot and numbers of larvae per lawn were counted after 24 hours.

Total no. of L1s added	No. on OP50-1 (%)	No. on JUb275(%)	No. elsewhere on plate
392	201 (51.2%)	148 (37.75%)	43 (10.7%)
