# Supplementary material for: The golden death bacillus Chryseobacterium nematophagum is a novel matrix digesting pathogen of nematodes
Source: BMC Biol. 2019 Feb 28;17:10. doi: 10.1186/s12915-019-0632-x (PMC6394051; doi:10.1186/s12915-019-0632-x)
Supplement: Supplementary file 6 — Killing of larval parasitic nematode species on exposure to Chryseobacterium nematophagum. (PDF 9871 kb) [file 12915_2019_632_MOESM6_ESM.pdf]

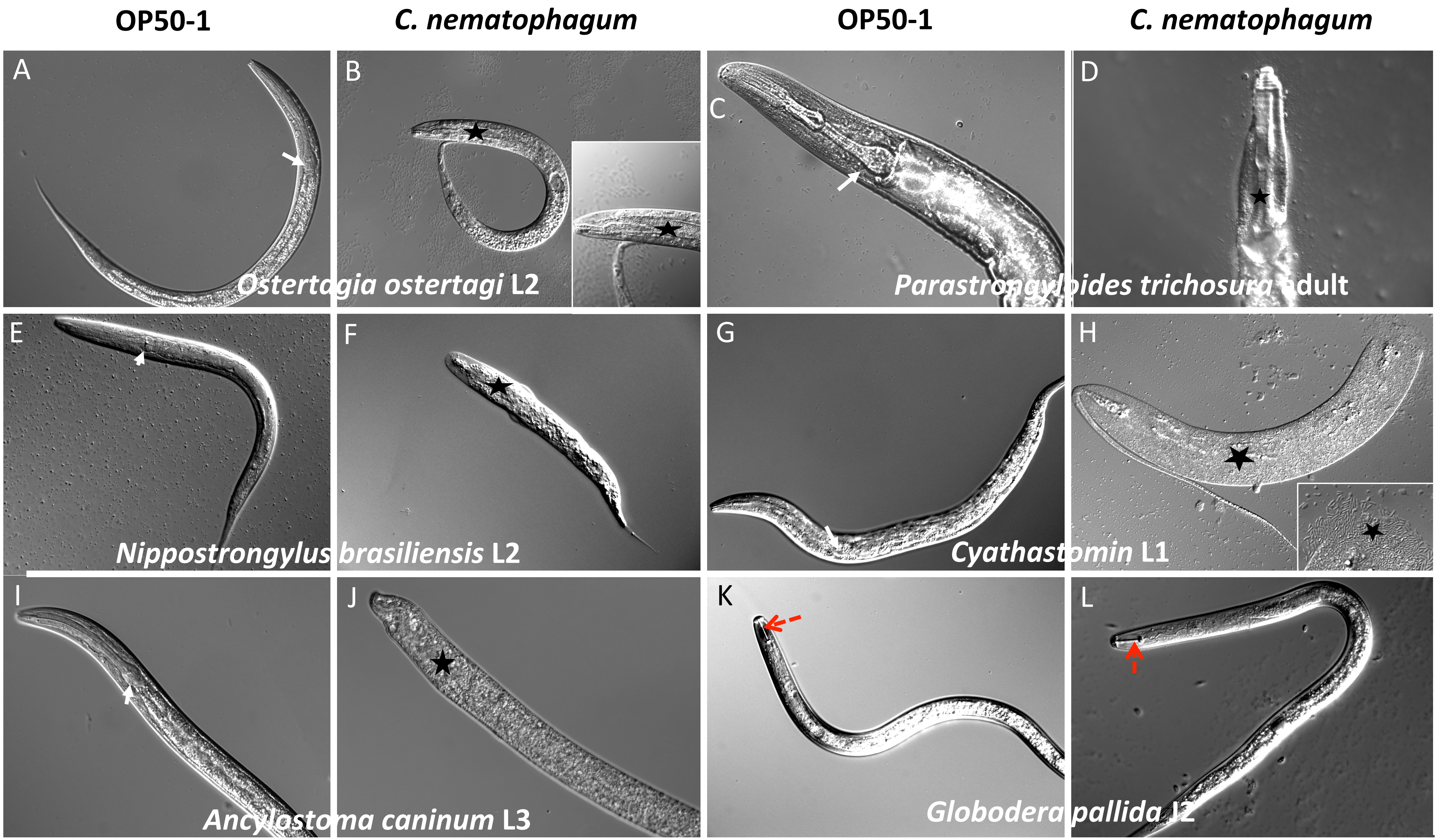

OP50-1
C. nematophagum
OP50-1
C. nematophagum
A
B
C
D
Ostertagia ostertagi L2
Parastrongyloides trichosura adult
E
F
G
H
Nippostrongylus brasiliensis L2
Cyathastomin L1
I
J
K
L
Ancylostoma caninum L3
Globodera pallida J2

Killing of parasitic nematode species by *Chryseobacterium nematophagum*

A. *Ostertagia ostertagi* (cattle) L2 with OP50, **B.** *O. ostertagi* L1 with *C. nematophagum* (inset x640)*,* **C.** *Parastrongyloides trichosura* (opossum) adult with OP50, **D.** *P. trichosura* adult with *C. nematophagum*, **E.** *Nippostrongylus brasiliensis* (rat) L2 with OP50, **H.** *N. brasiliensis* L1 with *C. nematophagum*, **G.** *Cyathastomin* (horse) L1 with OP50, **H.** *Cyathastomin* L1 (x640) with *C. nematophagum* (inset x1000), **I.** *Ancylostoma caninum* (wolf) L3 with OP50, **J.** *A. caninum* L3 with *C. nematophagum,* **K.** *Globodera pallida* (potato) J2 with OP50, **L.** G. pallida J2 with *C. nematophagum* (broken red arrow, stylet). All images at x250 magnification, except G which is at x 640 magnification. White arrow depicts pharyngeal bulb and black star highlights bacilli.
