# Supplementary material for: The golden death bacillus Chryseobacterium nematophagum is a novel matrix digesting pathogen of nematodes
Source: BMC Biol. 2019 Feb 28;17:10. doi: 10.1186/s12915-019-0632-x (PMC6394051; doi:10.1186/s12915-019-0632-x)
Supplement: Supplementary file 7 — Testing of Chryseobacterium nematophagum on mosquito larvae. (PDF 104 kb) [file 12915_2019_632_MOESM7_ESM.pdf]

AF Testing of *Chryseobacterium nematophagum* on mosquito larvae

Pots containing 50 ml water, dried yeast extract and approximately 200 freshly hatched *Aedes aegypti* (Liverpool strain) mosquito larvae were supplemented with 1 ml overnight bacterial inoculum. Larvae were cultured (26 °C) for 48 hrs then assessed for viability.

Figure. Images of larvae after 24 hours (X40), supplemented with A. OP50-1, B. JUb275 and C. JUb129.

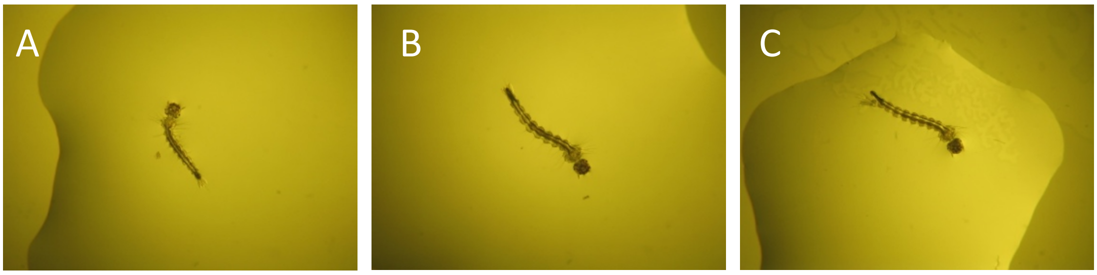

Assessment of Viability after 48 hr exposure

Bacteria	No. of live larvae	No. of dead larvae
**OP50-1**	180	6
**JUb129**	204	0
**JUB275**	185	0
