# Supplementary material for: The golden death bacillus Chryseobacterium nematophagum is a novel matrix digesting pathogen of nematodes
Source: BMC Biol. 2019 Feb 28;17:10. doi: 10.1186/s12915-019-0632-x (PMC6394051; doi:10.1186/s12915-019-0632-x)
Supplement: Supplementary file 12 — Distribution of PorS genes in the Chryseobacterium nematophagum genome. (PDF 401 kb) [file 12915_2019_632_MOESM12_ESM.pdf]

Distribution of PorS genes in the *C. nematophagum* (JUb129) genome

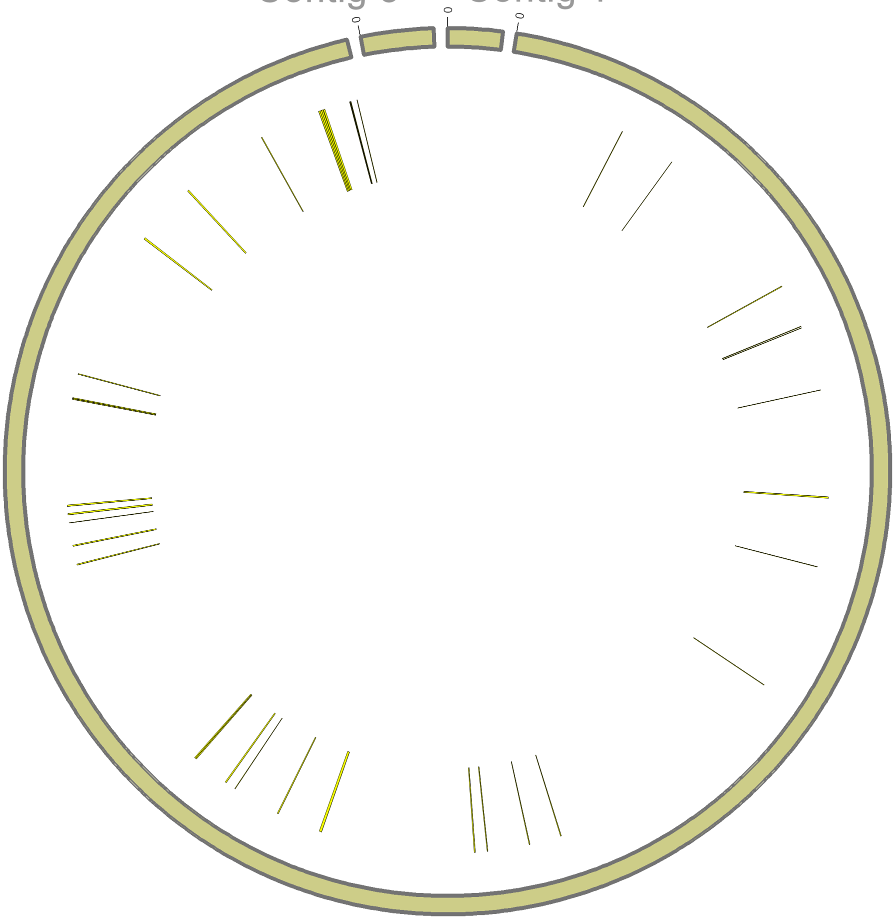

PorS genes are represented by 28 Hierarchical Orthologous Groups (HOGs), comprising 38 loci, which are distributed throughout the bacterial genome.

HOG01173_1	HOG01568_4	HOG04349_1	HOG04639
HOG01173_2	HOG01568_5	HOG04349_2	HOG04640
HOG01173_3	HOG01568_6	HOG04484	HOG04648
HOG01173_4	HOG04273	HOG04495	HOG03701_1
HOG01173_5	HOG04285	HOG04536	HOG03701_2
HOG01173_6	HOG04319	HOG04541	HOG03710_1
HOG01568_1	HOG04320	HOG04542	HOG03710_2
HOG01568_2	HOG04334	HOG04570	HOG03710_3
HOG01568_3	HOG04340	HOG04636	HOG04462
		HOG04638	HOG04531
