# Supplementary material for: The golden death bacillus Chryseobacterium nematophagum is a novel matrix digesting pathogen of nematodes
Source: BMC Biol. 2019 Feb 28;17:10. doi: 10.1186/s12915-019-0632-x (PMC6394051; doi:10.1186/s12915-019-0632-x)
Supplement: Supplementary file 13 — Chryseobacterium spp. genomes available in NCBI Genome database. (PDF 54 kb) [file 12915_2019_632_MOESM13_ESM.pdf]

Chryseobacterium spp. genomes available in NCBI Genome database

Accession code	Species
GCA_000143785.1	*Chryseobacterium gleum* ATCC 35910
GCA_000282115.1	*Chryseobacterium sp.* CF314
GCA_000520835.1	*Chryseobacterium indologenes* NBRC 14944
GCA_000729985.1	*Chryseobacterium antarcticum*
GCA_000735105.1	*Chryseobacterium halperniae*
GCA_000735135.1	*Chryseobacterium sp.* FH1
GCA_000735695.2	*Chryseobacterium haifense* DSM 19056
GCA_000737665.1	*Chryseobacterium formosense*
GCA_000737705.1	*Chryseobacterium soli*
GCA_000737715.1	*Chryseobacterium sp.* JM1
GCA_000737765.1	*Chryseobacterium vrystaatense*
GCA_000737775.1	*Chryseobacterium piperi*
GCA_000737785.1	*Chryseobacterium luteum*
GCA_000738495.1	*Chryseobacterium sp.* P1−3
GCA_000812865.1	*Chryseobacterium jeonii*
GCA_000812875.1	*Chryseobacterium solincola*
GCA_000813825.1	*Chryseobacterium taiwanense*
GCA_000820645.1	*Chryseobacterium oranimense* G311
GCA_000829375.1	*Chryseobacterium sp.* StRB126
GCA_001021975.1	*Chryseobacterium gallinarum*
GCA_001045435.1	*Chryseobacterium koreense* CCUG 49689
GCA_001045445.1	*Chryseobacterium sp.* BLS98
GCA_001045455.1	*Chryseobacterium sp.* FH2
GCA_001045465.1	*Chryseobacterium angstadtii*
GCA_001187685.1	*Chryseobacterium sp.* Hurlbut01
GCA_001295265.1	*Chryseobacterium indologenes*
GCA_001297705.1	*Chryseobacterium sp.* ERMR1:04
GCA_001420285.1	*Chryseobacterium aquaticum*
GCA_001421435.1	*Chryseobacterium sp.* Leaf201
GCA_001424105.1	*Chryseobacterium sp.* Leaf394
GCA_001424145.1	*Chryseobacterium sp.* Leaf404
GCA_001424585.1	*Chryseobacterium sp.* Leaf180
GCA_001425355.1	*Chryseobacterium sp.* Leaf405
GCA_001456155.1	*Chryseobacterium sp.* IHB B 17019
GCA_001507325.1	*Chryseobacterium greenlandense*
GCA_001507335.1	*Chryseobacterium sp.* JAH

GCA_001563495.1	*Chryseobacterium kwangjuense*
GCA_001593385.1	*Chryseobacterium cucumeris*
GCA_001643375.1	*Chryseobacterium sp.* FP211-J200
GCA_001648155.1	*Chryseobacterium sp.* IHBB 10212
GCA_001677935.1	*Chryseobacterium sp.* MOF25P
GCA_001677955.1	*Chryseobacterium sp.* BGARF1
GCA_001684955.1	*Chryseobacterium contaminans*
GCA_001684965.1	*Chryseobacterium arthrosphaerae*
GCA_001684975.1	*Chryseobacterium artocarpi*
GCA_001693475.1	*Chryseobacterium sp.* CBo1
GCA_001724115.1	*Chryseobacterium sp.* SCN 40-13
GCA_001898255.1	*Chryseobacterium sp.* 39-10
GCA_001898785.1	*Chryseobacterium sp.* 36-9
GCA_002177115.1	*Chryseobacterium mucoviscidosis*
GCA_002205795.1	*Chryseobacterium sp.* VAUSW3
GCA_002208925.2	*Chryseobacterium indologenes*
GCA_002216065.1	*Chryseobacterium sp.* T16E-39
GCA_002285635.2	*Chryseobacterium piperi*
GCA_002591275.1	*Chryseobacterium indologenes*
GCA_002754245.1	*Chryseobacterium sp.* 52
GCA_002797535.1	*Chryseobacterium geocarposphaerae*
GCA_002835665.1	*Chryseobacterium sp.* PMSZPI
GCA_002899825.2	*Chryseobacterium sp.* ISE14
GCA_002899875.1	*Chryseobacterium lactis*
GCA_002899895.2	*Chryseobacterium oncorhynchi*
GCA_002899945.2	*Chryseobacterium viscerum*
GCA_002943655.1	*Chryseobacterium shigense*
GCA_002943675.1	*Chryseobacterium piscicola*
GCA_002979455.1	*Chryseobacterium sp.* MYb7
GCA_002979665.1	*Chryseobacterium culicis*
GCA_002979755.1	*Chryseobacterium culicis*
GCA_003061225.1	*Chryseobacterium sp.* HMWF001
GCA_003061395.1	*Chryseobacterium sp.* HMWF028
GCA_003094695.1	*Chryseobacterium sp.* HMWF035
GCA_003182335.1	*Chryseobacterium sp.* AG844
GCA_003201365.1	*Chryseobacterium sp.* CBTAP 102
GCA_003248465.1	*Chryseobacterium sp.*
GCA_003248755.1	*Chryseobacterium sp.*

GCA_003336205.1	*Chryseobacterium sp.* YLOS41
GCA_900099685.1	*Chryseobacterium taeanense*
GCA_900100075.1	*Chryseobacterium jejuense*
GCA_900100115.1	*Chryseobacterium soldanellicola*
GCA_900102265.1	*Chryseobacterium hungaricum*
GCA_900103755.1	*Chryseobacterium taihuense*
GCA_900108025.1	*Chryseobacterium humi*
GCA_900108115.1	*Chryseobacterium bovis* DSM 19482
GCA_900108365.1	*Chryseobacterium culicis*
GCA_900108525.1	*Chryseobacterium hominis*
GCA_900109615.1	*Chryseobacterium antarcticum*
GCA_900109935.1	*Chryseobacterium taichungense*
GCA_900110605.1	*Chryseobacterium halperniae*
GCA_900111495.1	*Chryseobacterium wanjuense*
GCA_900112115.1	*Chryseobacterium jeonii*
GCA_900113805.1	*Chryseobacterium frigidisoli*
GCA_900113975.1	*Chryseobacterium indologenes*
GCA_900114045.1	*Chryseobacterium treverense*
GCA_900114875.1	*Chryseobacterium limigenitum*
GCA_900115055.1	*Chryseobacterium oleae*
GCA_900116415.1	*Chryseobacterium formosense*
GCA_900128945.1	*Chryseobacterium sp.* OV279
GCA_900129105.1	*Chryseobacterium sp.* YR203
GCA_900129245.1	*Chryseobacterium arachidis*
GCA_900129385.1	*Chryseobacterium takakiae*
GCA_900129755.1	*Chryseobacterium oranimense*
GCA_900141765.1	*Chryseobacterium zeae*
GCA_900142325.1	*Chryseobacterium molle*
GCA_900142445.1	*Chryseobacterium polytrichastri*
GCA_900142615.1	*Chryseobacterium contaminans*
GCA_900142785.1	*Chryseobacterium carnipullorum*
GCA_900143185.1	*Chryseobacterium scophthalmum*
GCA_900156015.1	*Chryseobacterium sp.* RU33C
GCA_900156075.1	*Chryseobacterium sp.* RU37D
GCA_900156145.1	*Chryseobacterium indoltheticum*
GCA_900156575.1	*Chryseobacterium shigense*
GCA_900156585.1	*Chryseobacterium joostei*
GCA_900156685.1	*Chryseobacterium piscicola*

GCA_900156725.1	*Chryseobacterium chaponense*
GCA_900156735.1	*Chryseobacterium ureilyticum*
GCA_900156825.1	*Chryseobacterium gambrini*
GCA_900168205.1	*Chryseobacterium balustinum*
GCA_900176315.1	*Chryseobacterium sp.* YR221
GCA_900187185.1	*Chryseobacterium taklimakanense*
GCA_900446785.1	*Chryseobacterium balustinum*
GCA_900446855.1	*Chryseobacterium jejuense*
